# Membrane anatomy-guided robot-assisted partial nephrectomy: operational definition and association with warm ischemia time in a single-surgeon cohort

**DOI:** 10.3389/fonc.2026.1953453

**Published:** 2026-09-24

**Authors:** Wenhao Chu, Songbai Yan, Mengying Liao, Rui Hu, Yu Yang, Mingjie He, Guanqun Wang, Liangkuan Bi, Xing Fu

**Affiliations:** 1 Department of Urology, Peking University Shenzhen Hospital, Shenzhen, China; 2 Shenzhen Key Laboratory of Male Reproductive Medicine and Genetics, Institute of Urology, Peking University Shenzhen Hospital, Shenzhen, China; 3 Shenzhen Clinical Research Center for Urology and Nephrology, Peking University Shenzhen Hospital,, Shenzhen, China; 4 Department of Pathology, Peking University Shenzhen Hospital, Shenzhen, China

**Keywords:** fascial plane, membrane anatomy, robot-assisted partial nephrectomy, surgical efficiency, tumor enucleation, warm ischemia time

## Abstract

**Background:**

A standardized anatomical framework for robot-assisted partial nephrectomy (RAPN) is lacking. This study evaluates a stepwise membrane anatomy-guided pathway and its association with warm ischemia time (WIT) and clamping-phase efficiency, controlling for surgical experience and temporal confounding.

**Methods:**

We evaluated 151 consecutive RAPN cases (30 conventional vs. 121 membrane anatomy-guided) performed by a single-center, single-surgeon cohort using the da Vinci Xi System (2022–2025). Multivariable linear regression with HC3 robust standard errors was performed to assess the primary outcome (WIT), adjusting for clinical covariates and nonlinear cumulative case sequence (natural cubic spline, df = 3). Chronology-adjusted sensitivity analyses and propensity-score overlap weighting were conducted.

**Results:**

Despite substantial chronological overlap between pathways, the membrane-guided pathway was independently associated with a 4.05-min reduction in mean WIT (95% CI -6.82 to -1.28; *P* = 0.004) without compromising surgical margin safety. This effect was consistent across linear, calendar-time, propensity-weighted, and overlap-cohort sensitivity analyses (-3.67 to -4.19 min; all *P* < 0.05). The membrane-guided approach also significantly increased the odds of WIT ≤ 25 min (adjusted OR 5.25; *P* = 0.003) and modified Trifecta achievement (adjusted OR 4.97; *P* = 0.005).

**Conclusion:**

The membrane anatomy-guided pathway consistently improves clamping-phase efficiency and shortens WIT in RAPN after accounting for learning curves and temporal trends. Given the retrospective design, these findings reflect associative rather than definitive causal effects.

## Introduction

Partial nephrectomy is a cornerstone treatment for localized renal tumors (1). Current renal cell carcinoma guidelines emphasize balancing oncological control, preservation of renal function, and perioperative safety (1). In robot-assisted partial nephrectomy (RAPN), warm ischemia time (WIT) is an important marker of procedural performance during renal hilar clamping. However, its clinical significance should not be reduced to a simple “shorter is always better” principle or interpreted in isolation as equivalent to long-term renal functional benefit (2). Current evidence suggests that the relationship between WIT and renal outcomes depends on multiple factors, while extreme attempts to avoid clamping may increase technical complexity and bleeding risk (2). Accordingly, reducing avoidable time within a controlled clamping interval may be better regarded as an indicator of procedural efficiency and operative standardization.

The concept of membrane anatomy originated from reproducible fascial planes in rectal surgery and later expanded to abdominopelvic surgery (3–5). Subsequent work further standardized its terminology and classification (6). In partial nephrectomy, the tumor pseudocapsule is relevant to enucleation (7, 8). Retroperitoneal fascial anatomy relevant to surgical-plane definition has been described in anatomical and imaging studies (9, 10). Related membrane-anatomy frameworks have also been discussed in urological surgery (11–13). Organizing these landmarks into a predefined operative framework may permit selected navigation and preparation steps to be completed before arterial clamping, thereby reducing non-essential activity during the ischemic interval.

We therefore sought to operationally define a membrane anatomy-guided pathway for RAPN and evaluate its association with WIT and clamping-phase efficiency in a single-surgeon cohort. Because the membrane anatomy-guided concept was gradually incorporated into clinical practice rather than implemented at a single transition point, particular attention was given to cumulative operative experience and calendar-time confounding. We additionally examined whether the observed association remained similar across complementary chronology-adjusted, propensity-weighted, and concurrent-period analyses.

## Patients and methods

### Patients and study design

This single-center, single-surgeon, retrospective phased cohort study analyzed 342 consecutive patients undergoing minimally invasive partial nephrectomy at Peking University Shenzhen Hospital between December 2022 and August 2025. The primary analytical cohort comprised 151 RAPN cases performed using the da Vinci Xi Surgical System, categorized into conventional (n = 30) and membrane anatomy-guided (n = 121) pathway groups. The remaining 191 laparoscopic partial nephrectomy (LPN) cases were reserved for secondary exploratory analyses.

The membrane anatomy-guided pathway was introduced gradually into clinical practice rather than through an abrupt protocol transition, resulting in substantial chronological overlap between the two pathways. To protect patient privacy and rigorously account for cumulative surgical experience, chronology was modeled using cumulative case sequence rather than calendar dates.

The study was approved by the Ethics Committee of Peking University Shenzhen Hospital (approval No. 2026144). The requirement for written informed consent was waived due to the retrospective design and use of anonymized clinical data.

### Exposure ascertainment and operational definition

Operative pathways were extracted directly from contemporaneous surgical records rather than assigned based on warm ischemia time, perioperative outcomes, operation date, or study findings. Procedures were classified into two groups: the conventional pathway group, characterized by routine tissue dissection without a specific membrane-anatomical interface, and the membrane anatomy-guided pathway, which utilized identifiable, nameable, and reproducibly accessible fascial or pseudocapsular interfaces as intraoperative navigation landmarks (4).

The central concept of this membrane anatomy-guided pathway is to complete plane identification, hilar preparation, and resection-boundary judgement as far as possible before arterial clamping (2). The pathway included preoperative imaging-based assessment and four sequential intraoperative technical nodes, integrated into a standardized conceptual workflow (**Figure 1A**):

**Figure 1:**
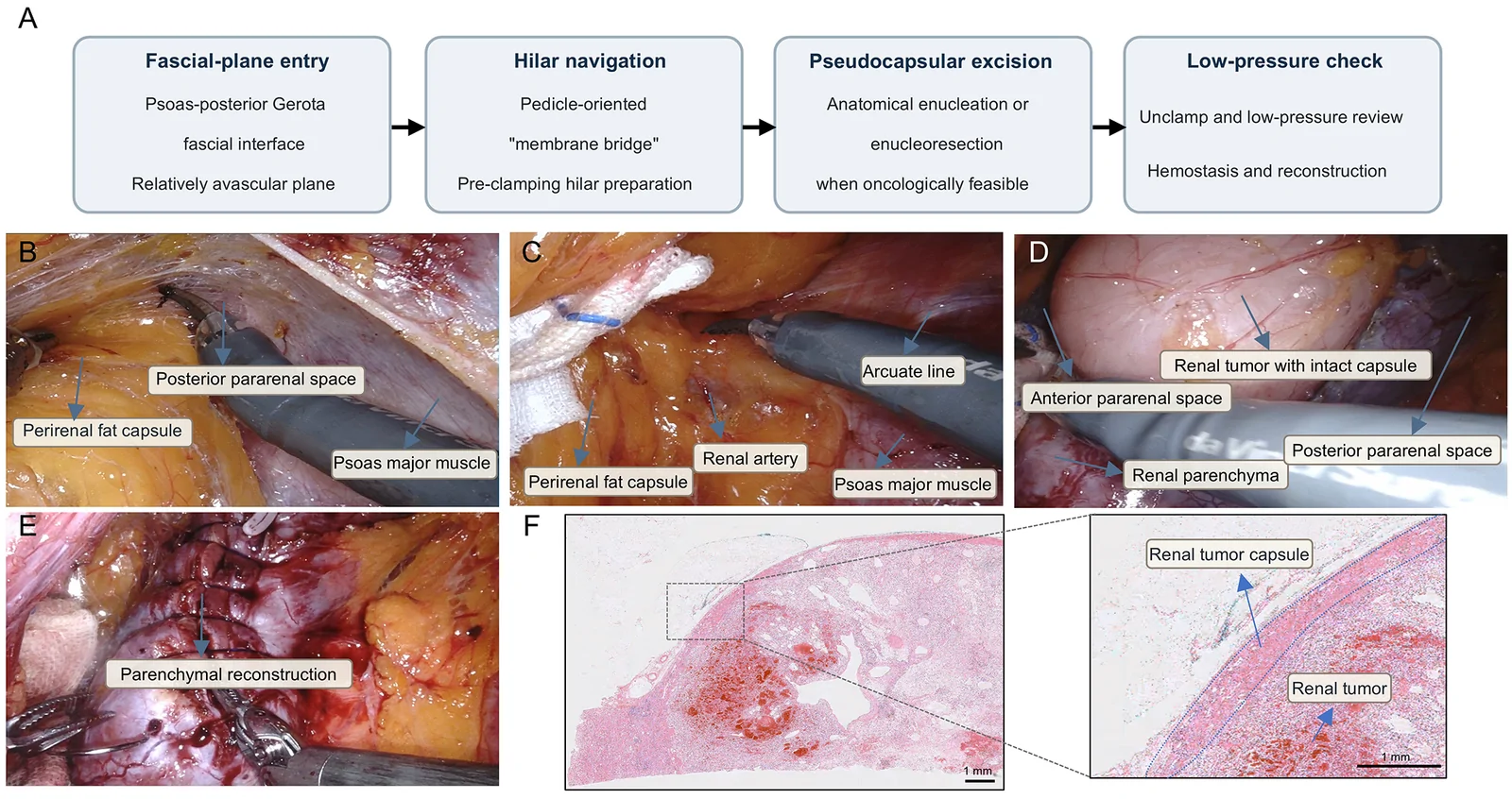
Operational framework of the membrane anatomy-guided pathway for robot-assisted partial nephrectomy (RAPN). **(A)** Schematic representation of the four sequential technical nodes: fascial-plane entry, hilar navigation, pseudocapsular excision, and low-pressure check. **(B)** Fascial-plane entry showing the perirenal fat capsule, posterior pararenal space, and psoas major muscle within the avascular plane. **(C)** Hilar navigation with identification of the main renal artery along the pedicle-oriented interface before arterial clamping. **(D)** Intraoperative identification of the renal tumor and surrounding anatomical planes prior to pseudocapsule-guided excision. **(E)** Completed parenchymal reconstruction after tumor excision prior to low-pressure inspection and unclamping [conceptualized in **(A)**]. **(F)** Representative hematoxylin-and-eosin-stained section (scale bars = 1 mm) demonstrating the fibrous renal tumor pseudocapsular boundary.

1. Fascial-plane entry: After incising Gerota**’**s fascia, the surgeon precisely identifies and enters the relatively avascular potential space between the perirenal fascia and the fusion fascia based on established retroperitoneal and urogenital fascial anatomy (**Figure 1B**) (9, 10).
2. Hilar navigation via membrane bridge: The surgeon dissects along an anatomical bridge at the level of the hilar pedicle plate or vascular sheath, skeletonizing the main renal artery or the intended branch for clamping (**Figure 1C**) (11–13).
3. Pseudocapsule-guided excision: After controlled arterial clamping, the surgeon uses the fibrous tumor pseudocapsule as a natural, oncologically safe boundary to perform precise extracapsular enucleation or enucleoresection under high-definition three-dimensional visualization (**Figure 1D**) (7, 8).
4. Low-pressure check Following completed parenchymal reconstruction (**Figure 1E**) and restoration of blood flow, pneumoperitoneum pressure is reduced to 6 to 8 mmHg to dynamically review and confirm hemostatic and repair quality at the resection bed and renal hilum. Bleeding identified specifically during this low-pressure inspection was not prospectively captured as a separate study variable.

In retroperitoneal RAPN, for example, the surgeon entered the relatively loose hypovascular plane adjacent to the posterior layer of Gerota’s fascia and subsequently approached the renal hilum along the pedicle-plate or vascular-sheath interface. During tumor excision, the interface between the pseudocapsule and adjacent renal parenchyma served as the principal resection boundary. Representative histopathological sections demonstrated a thin fibrous boundary corresponding to the operative pseudocapsular plane (**Figure 1F**).

These technical nodes were used to standardize the methodological description of the membrane anatomy-guided concept and were not retrospectively scored to redefine pathway assignment in the present analysis.

### Outcomes

The primary outcome was WIT, defined as the duration of renal arterial clamping.

Secondary perioperative outcomes included operative time, estimated blood loss, postoperative length of stay, drain removal time, Clavien-Dindo complication grade, positive surgical margin, postoperative change in estimated glomerular filtration rate (eGFR), and hemoglobin change.

Two supportive secondary efficiency endpoints were evaluated: achievement of WIT ≤ 25 min and modified Trifecta. Modified Trifecta was defined as fulfillment of all three criteria: WIT ≤ 25 min, negative surgical margins, and absence of Clavien-Dindo grade ≥ III complications.

### Statistical analysis

For descriptive statistics, continuous variables were expressed as mean ± standard deviation or median with interquartile range [IQR], depending on distribution, and compared using Welch’s t-test or the Mann-Whitney U test. Categorical variables were expressed as frequencies with percentages and compared using Pearson’s chi-square test or Fisher’s exact test. Baseline balance was assessed using standardized mean differences (SMDs), with an absolute SMD > 0.10 indicating meaningful imbalance.

To rigorously control for confounding bias, a multi-level statistical framework was constructed. The primary analysis used multivariable linear regression with HC3 robust standard errors to evaluate the independent association between surgical pathway and WIT, adjusting for clinical covariates—including age, sex, body mass index (BMI), tumor maximum diameter, baseline eGFR, RENAL (Radius, Exophytic/Endophytic, Nearness, Anterior/Posterior, Location) score, Mayo Adhesive Probability (MAP) score, surgical approach, and intraoperative indocyanine green (ICG) use—and cumulative RAPN case sequence modeled via a natural cubic regression spline (with 3 degrees of freedom).

To rigorously test the stability of the primary association against residual temporal and surgical-experience confounding, multiple complementary sensitivity analyses were conducted. First, cumulative case sequence was modeled as a linear continuous variable, and alternatively replaced by calendar-time natural cubic splines (3 degrees of freedom). Second, adjusted median regression was performed using the same clinical covariates and spline term, with 95% confidence intervals estimated via 2,000 patient-level nonparametric bootstrap resamples. Third, propensity-score overlap weighting incorporating clinical covariates and the case-sequence spline was evaluated, examining effective sample size, weight distributions, extreme weights, and post-weighting SMDs. Fourth, direct chronological comparability was assessed by restricting the primary model to the calendar-overlap cohort (cases 9–149) and by applying 1:1 temporally adjacent matching using a preferred 30-day maximum temporal caliper. Finally, an unadjusted cumulative sum (CUSUM) curve for WIT was constructed descriptively to visualize temporal performance evolution.

Supportive binary outcomes (WIT ≤ 25 min and modified Trifecta) were analyzed using multivariable logistic regression adjusting for clinical covariates and the case-sequence spline. In the broader 342-case minimally invasive cohort, surgical platform and chronology were accounted for in exploratory sensitivity models, including an exploratory pathway-by-platform interaction term.

All analyses used complete observations for variables included in the respective models without imputation or automated variable selection. Statistical tests were two-sided, with *P* < 0.05 considered significant. Analyses were performed using Python 3.13.14 (statsmodels 0.14.6 and Patsy 1.0.2).

## Results

### Cohort composition and chronological distribution

A total of 151 patients met the inclusion criteria for the primary RAPN cohort, comprising 30 conventional and 121 membrane anatomy-guided procedures. Adoption of the membrane anatomy-guided concept was gradual rather than defined by an abrupt transition. The first membrane-guided RAPN occurred at cumulative case 9, whereas conventional procedures continued up to cumulative case 149. Overall, 22 of 30 conventional procedures occurred after the first membrane-guided RAPN, and 119 of 121 membrane-guided procedures occurred before the last conventional RAPN.

To protect patient privacy, surgical chronology was described using cumulative RAPN case sequence rather than calendar dates. A descriptive plot of WIT against cumulative case sequence illustrates this chronological overlap, alongside the overall temporal trajectory of surgical performance fitted via a LOWESS curve (**Figure 2**).

**Figure 2:**
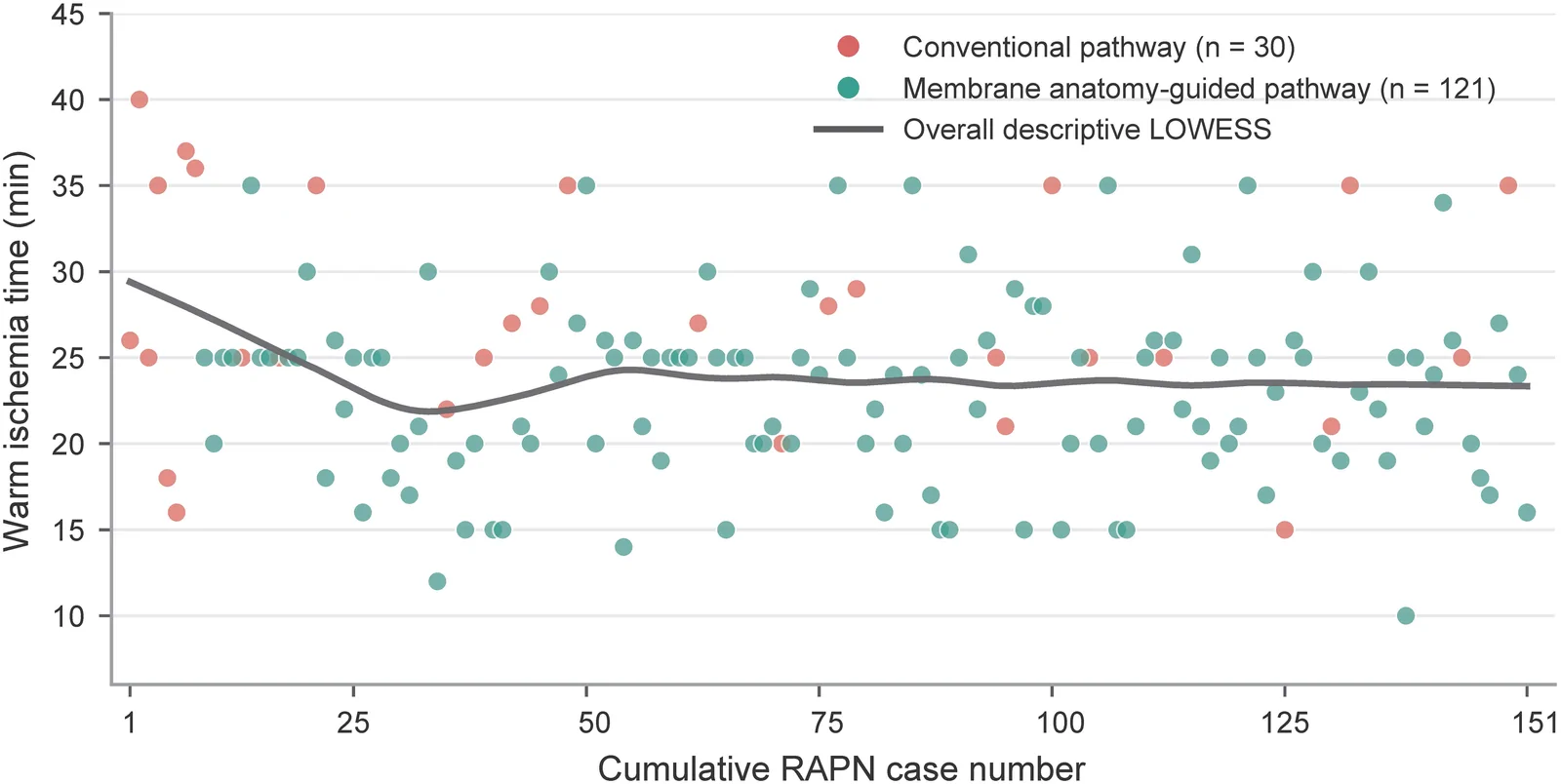
Warm ischemia time (WIT) across cumulative case sequence in the robot-assisted partial nephrectomy (RAPN) cohort. WIT is plotted against the cumulative RAPN case sequence (cases 1 to 151), with individual procedures colored by operative pathway: conventional pathway (pink circles, n = 30) and membrane anatomy-guided pathway (green circles, n = 121). The solid grey LOWESS curve illustrates the overall descriptive temporal trajectory of warm ischemia time across cumulative operative experience and is not intended for statistical inference or to define a discrete learning-curve cutoff.

### Baseline characteristics

Baseline demographic, clinical, tumor, and chronological characteristics are shown in **Table 1**. Unadjusted comparisons showed no significant differences between the two pathways regarding key clinical parameters (including age, sex, BMI, tumor maximum diameter, preoperative eGFR, and RENAL score; all *P* > 0.05), with the exception of MAP score (*P* = 0.010) and cumulative RAPN case number (*P* = 0.017).

**Table 1:** Baseline demographic, clinical, tumor, and chronological characteristics of the primary robot-assisted partial nephrectomy (RAPN) cohort according to operative pathway.

Characteristic	Data type	Overall (n = 151)	Conventional pathway (n = 30)	Membrane anatomy-guided pathway (n = 121)	*P*	Unweighted SMD	Statistical test
Age, years	Mean ± SD	51.66 ± 14.10	50.90 ± 15.55	51.84 ± 13.78	0.763	0.065	Welch’s t-test
Sex: male	n (%)	87 (57.6%)	14 (46.7%)	73 (60.3%)	0.175	0.277	Pearson chi-square test
BMI, kg/m^2^	Median [IQR]	24.30 [21.45, 26.85]	23.90 [22.10, 27.77]	24.40 [21.10, 26.70]	0.565	-0.085	Mann-Whitney U test
Tumor maximum diameter, cm	Median [IQR]	3.40 [2.50, 4.80]	3.30 [2.55, 4.45]	3.50 [2.50, 4.80]	0.974	-0.010	Mann-Whitney U test
Preoperative eGFR, mL/min/1.73 m²	Mean ± SD	96.95 ± 21.01	102.63 ± 23.55	95.54 ± 20.18	0.137	-0.327	Welch’s t-test
RENAL score	Median [IQR]	7.00 [6.00, 9.00]	8.00 [7.00, 9.00]	7.00 [6.00, 9.00]	0.153	-0.248	Mann-Whitney U test
MAP score	Median [IQR]	1.00 [0.00, 2.00]	1.50 [1.00, 2.00]	1.00 [0.00, 2.00]	0.010	-0.550	Mann-Whitney U test
Transperitoneal approach	n (%)	51 (33.8%)	9 (30.0%)	42 (34.7%)	0.625	0.101	Pearson chi-square test
ICG use	n (%)	53 (35.1%)	8 (26.7%)	45 (37.2%)	0.280	0.227	Pearson chi-square test
Cumulative RAPN case number	Median [IQR]	76.00 [38.50, 113.50]	46.50 [9.25, 98.75]	81.00 [46.00, 116.00]	0.017	0.472	Mann-Whitney U test

BMI, body mass index; eGFR, estimated glomerular filtration rate; ICG, indocyanine green; IQR, interquartile range; MAP, Mayo Adhesive Probability; RAPN, robot-assisted partial nephrectomy; RENAL, RENAL nephrometry score; SD, standard deviation; SMD, standardized mean difference. *P* < 0.05 are considered statistically significant.

However, evaluation of SMDs identified meaningful measured imbalance (|SMD| > 0.10) across several characteristics before adjustment, particularly MAP score and cumulative case number. Additional imbalance was observed for preoperative eGFR, sex, RENAL score, and ICG use (**Table 1**). These findings indicated that hypothesis testing alone did not establish baseline equivalence, supporting multivariable and propensity-weighted adjustments incorporating clinical and chronological factors.

### Unadjusted perioperative outcomes

As detailed in **Table 2**, implementation of the membrane anatomy-guided pathway demonstrated a pronounced advantage in clamping-phase efficiency during unadjusted comparisons. The primary outcome, median WIT, was significantly shorter in the membrane anatomy-guided group than in the conventional group (24.0 min [IQR 20.0–25.0] vs. 25.5 min [IQR 25.0–35.0], *P* < 0.001; **Figure 3**). This translated into superior efficiency profiles, with markedly higher proportions of patients achieving WIT ≤ 25 min (76.0% vs. 50.0%, *P* = 0.005) and modified Trifecta (74.4% vs. 50.0%, *P* = 0.009).

**Table 2:** Perioperative outcomes and modified Trifecta achievement in the primary robot-assisted partial nephrectomy (RAPN) cohort.

Outcome	Data type	Overall (n = 151)	Conventional pathway (n = 30)	Membrane anatomy-guided pathway (n = 121)	*P*	Unweighted SMD	Statistical test
Operative time, min	Median [IQR]	150.00 [120.00, 180.00]	150.00 [120.00, 175.00]	155.00 [120.00, 185.00]	0.460	0.106	Mann-Whitney U test
WIT, min	Median [IQR]	25.00 [20.00, 26.00]	25.50 [25.00, 35.00]	24.00 [20.00, 25.00]	< 0.001	-0.741	Mann-Whitney U test
Estimated blood loss, mL	Median [IQR]	20.00 [20.00, 50.00]	20.00 [20.00, 45.00]	20.00 [20.00, 50.00]	0.798	0.119	Mann-Whitney U test
Postoperative length of stay, days	Median [IQR]	5.00 [5.00, 7.00]	6.00 [5.00, 7.00]	5.00 [5.00, 6.00]	0.049	-0.185	Mann-Whitney U test
Drain removal time, days	Median [IQR]	4.00 [3.00, 5.00]	4.00 [3.00, 6.00]	4.00 [3.00, 5.00]	0.262	-0.082	Mann-Whitney U test
Postoperative eGFR change, mL/min/1.73 m²	Median [IQR]	-5.60 [-11.05, -0.30]	-7.10 [-9.45, -1.82]	-5.40 [-11.10, 2.70]	0.629	0.096	Mann-Whitney U test
Hemoglobin change, g/L	Median [IQR]	-10.00 [-15.00, -5.00]	-8.00 [-14.50, -4.00]	-11.00 [-15.00, -5.00]	0.306	-0.032	Mann-Whitney U test
WIT ≤ 25 min	n (%)	107 (70.9%)	15 (50.0%)	92 (76.0%)	0.005	0.560	Pearson chi-square test
Positive surgical margin	n (%)	2 (1.3%)	0 (0.0%)	2 (1.7%)	1.000	0.183	Fisher’s exact test
Clavien-Dindo grade ≥ III	n (%)	3 (2.0%)	0 (0.0%)	3 (2.5%)	1.000	0.225	Fisher’s exact test
Modified Trifecta	n (%)	105 (69.5%)	15 (50.0%)	90 (74.4%)	0.009	0.519	Pearson chi-square test

eGFR, estimated glomerular filtration rate; IQR, interquartile range; RAPN, robot-assisted partial nephrectomy; SMD, standardized mean difference; WIT, warm ischemia time. *P* < 0.05 are considered statistically significant.

**Figure 3:**
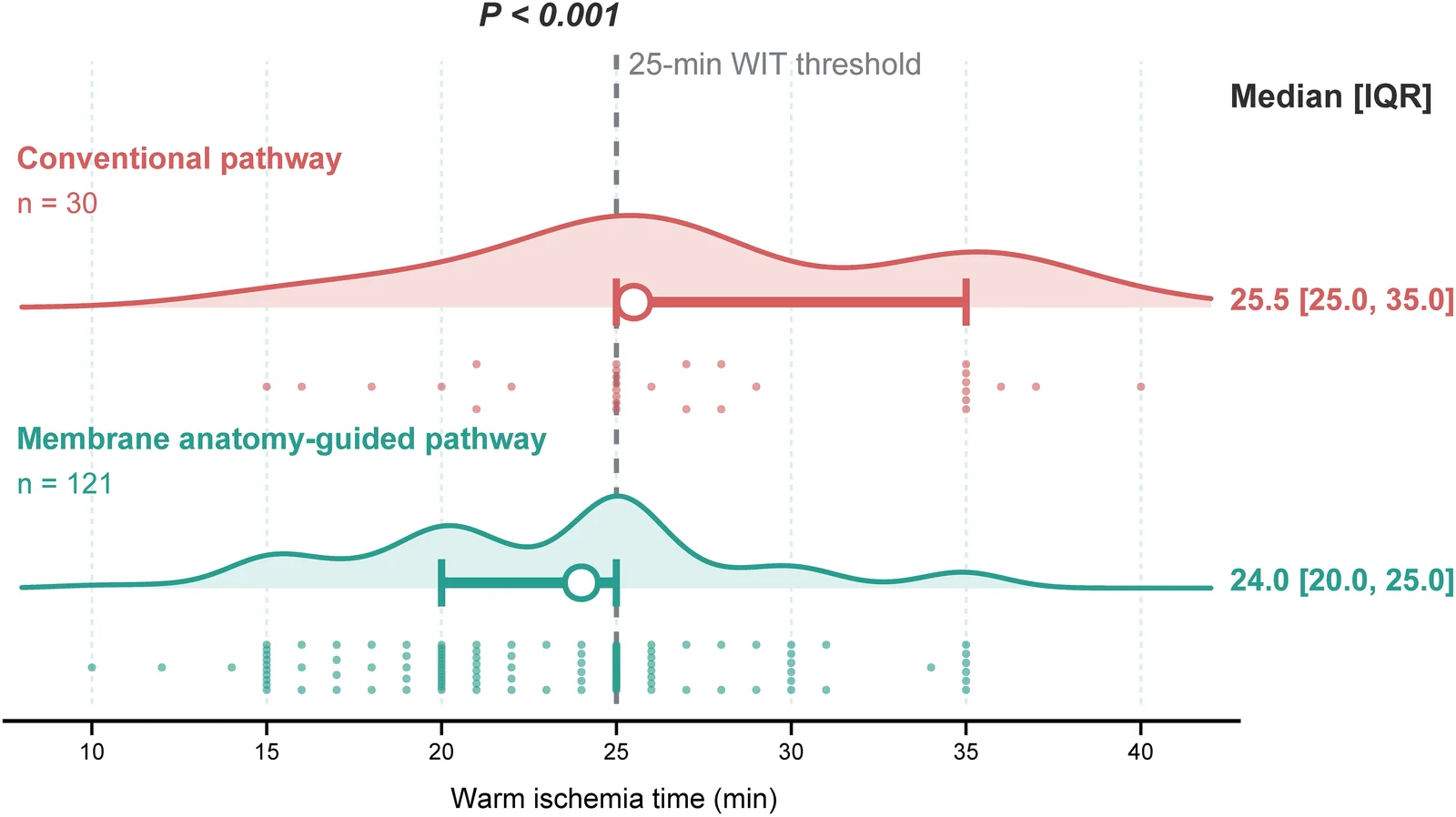
Distribution of warm ischemia time (WIT) according to operative pathway. The plot illustrates the kernel density estimations (ridge plots), individual patient scatter points, and median values with corresponding interquartile ranges (IQRs, horizontal error bars) for the conventional pathway (n = 30) and the membrane anatomy-guided pathway (n = 121) in the primary robot-assisted cohort. The vertical dashed line denotes the clinically relevant 25-min WIT quality-control threshold. The unadjusted comparison between groups was evaluated using the Mann–Whitney U test (*P* < 0.001). Corresponding descriptive statistics are presented in **Table 2**.

Parallel to these gains, postoperative length of stay was significantly reduced in the membrane anatomy-guided group (median 5.0 [IQR 5.0–6.0] vs. 6.0 [IQR 5.0–7.0] days, *P* = 0.049). Crucially, this optimization was achieved without compromising surgical safety or early functional preservation: total operative time, estimated blood loss, drain removal time, eGFR change, hemoglobin drop, major complications (Clavien-Dindo grade ≥ III), and positive surgical margin rates showed no significant differences between groups (all *P* > 0.05; **Table 2**).

### Primary and chronological sensitivity analyses

In the primary multivariable linear regression adjusting for clinical covariates and the nonlinear cumulative case sequence, the membrane anatomy-guided pathway was independently associated with a 4.05-min reduction in mean WIT compared with the conventional pathway (95% CI, -6.82 to -1.28; *P* = 0.004; **Table 3**). This was highly consistent with the baseline clinical-covariate model omitting case sequence (-4.29 min; 95% CI, -6.96 to -1.63; *P* = 0.002; **Table 3**).

**Table 3:** Primary and sensitivity analyses of the association between the membrane anatomy-guided pathway and warm ischemia time (WIT) in the primary robot-assisted partial nephrectomy (RAPN) cohort.

Analysis	Cohort	n	Difference in WIT (min)	95% CI	*P*	Chronology control
Unadjusted pathway comparison	Primary RAPN cohort	151	-4.38	-6.96 to -1.80	< 0.001	None
Original clinical-covariate adjusted HC3 model, no chronology	Primary RAPN cohort	151	-4.29	-6.96 to -1.63	0.002	None
PRIMARY: case-number spline + covariates, HC3	Primary RAPN cohort	151	-4.05	-6.82 to -1.28	0.004	Centered natural cubic spline of cumulative RAPN case number, df = 3
Linear case-number sensitivity	Primary RAPN cohort	151	-4.19	-6.85 to -1.53	0.002	Linear cumulative RAPN case number
Calendar-time spline sensitivity	Primary RAPN cohort	151	-3.67	-6.50 to -0.83	0.011	Centered natural cubic spline of calendar days from first RAPN, df = 3
Adjusted median regression	Primary RAPN cohort	151	-3.74	-7.72 to 0.14	0.065	Centered natural cubic spline of cumulative RAPN case number, df = 3
Chronology-informed overlap weighting	Primary RAPN cohort	151	-4.01	-6.87 to -1.16	0.006	PS centered natural cubic spline of cumulative RAPN case number, df = 3
Calendar-overlap cohort analysis	Calendar-overlap RAPN cohort	141	-4.14	-7.18 to -1.10	0.008	Centered natural cubic spline of cumulative RAPN case number, df = 3

CI, confidence interval; HC3, heteroskedasticity-consistent covariance estimator 3; PS, propensity score; RAPN, robot-assisted partial nephrectomy; WIT, warm ischemia time. Difference in WIT is calculated as membrane-guided minus conventional pathway. Models were adjusted for baseline clinical covariates and chronological sequence as indicated. *P* < 0.05 are considered statistically significant.

Multiple complementary sensitivity analyses confirmed the stability of this association across different chronological specifications (**Table 3**). The mean WIT reduction remained virtually identical when modeling cumulative experience as a linear variable (-4.19 min; 95% CI, -6.85 to -1.53; *P* = 0.002), utilizing a nonlinear calendar-time spline (-3.67 min; 95% CI, -6.50 to -0.83; *P* = 0.011), and restricting analysis to the calendar-overlap cohort (n = 141; -4.14 min; 95% CI, -7.18 to -1.10; *P* = 0.008).

Furthermore, propensity-score overlap weighting achieved excellent covariate balance (|SMD| ≤ 0.10 for all variables; **Supplementary Table S1**; **Supplementary Figures S1, S2**) and confirmed a significant 4.01-min reduction in mean WIT (95% CI, -6.87 to -1.16; *P* = 0.006; **Table 3**). Adjusted median regression yielded a directionally consistent estimate of -3.74 min (bootstrap 95% CI, -7.72 to 0.14; *P* = 0.065; **Table 3**).

### Secondary outcomes and exploratory analyses

In multivariable logistic regression adjusting for clinical covariates and cumulative case sequence, the membrane anatomy-guided pathway was associated with significantly higher odds of achieving WIT ≤ 25 min (adjusted OR, 5.25; 95% CI, 1.74 to 15.85; *P* = 0.003) and modified Trifecta (adjusted OR, 4.97; 95% CI, 1.63 to 15.13; *P* = 0.005; **Supplementary Table S2**). Positive surgical margins and major complications (Clavien-Dindo grade ≥ III) remained rare in both groups (**Supplementary Table S2**).

Chronological and learning dynamics were further supported by descriptive and matched analyses. The unadjusted CUSUM curve peaked at case 21 before steadily declining (**Supplementary Figure S3**). In the 1:1 temporally adjacent matched cohort (22 pairs; median absolute date difference: 1 day), the paired mean WIT difference was -4.05 min (95% CI, -7.41 to -0.68; *P* = 0.021; **Supplementary Table S3**).

In the expanded exploratory cohort of 342 minimally invasive partial nephrectomy cases, findings remained fully concordant: the membrane-guided approach yielded significant WIT reductions after adjusting for cumulative case sequence (-4.86 min; 95% CI, -6.98 to -2.74; *P* < 0.001) and calendar time (-4.37 min; 95% CI, -6.47 to -2.26; *P* < 0.001; **Supplementary Table S4**). A pathway-by-platform interaction model showed no significant effect modification between laparoscopic and robotic modalities (interaction: 0.34 min; 95% CI, -3.83 to 4.50; *P* = 0.874; **Supplementary Table S4**).

## Discussion

The principal finding of this study was that the membrane anatomy-guided operative pathway was associated with shorter WIT in a single-surgeon RAPN cohort (**Table 2**), with an effect magnitude that remained consistent after explicit adjustment for cumulative operative experience and calendar time (**Table 3**). In the primary chronology-adjusted model, the membrane-guided pathway was associated with a 4.05-min shorter mean WIT. Comparable estimates were obtained across linear, nonlinear calendar-time, propensity-score overlap-weighting, and concurrent-period sensitivity models. Although adjusted median regression yielded a directionally concordant estimate (-3.74 min) whose bootstrap 95% CI slightly crossed the null (-7.72 to 0.14 min, *P* = 0.065), likely reflecting reduced nonparametric power in skewed subgroup sizes, the effect magnitude closely aligned with the primary mean model (-4.05 min). Together, these findings support an independent association between the membrane anatomy-guided pathway and clamping-phase efficiency without establishing definitive causality.

A major consideration in interpreting this cohort is its chronological structure, as the membrane anatomy-guided concept was gradually incorporated into clinical practice rather than introduced at an abrupt transition point. Importantly, conventional and membrane-guided procedures were not simply separated into early and late eras: 22 of 30 conventional procedures occurred after the first membrane-guided RAPN, and 119 of 121 membrane-guided cases preceded the final conventional RAPN (**Figure 2**). The CUSUM trajectory peaked around case 21 (**Supplementary Figure S3**), capturing the initial exploratory phase after the first membrane-guided case (case 9) before plateauing into steady efficiency gains (**Figure 2**). To explicitly account for this learning curve, cumulative case sequence was modeled via nonlinear splines, and complementary calendar-time models yielded highly consistent estimates (**Table 3**). Furthermore, propensity-score overlap weighting achieved excellent balance across measured clinical and chronological covariates (|SMD| ≤ 0.10; **Tables 1**; **Supplementary Table S1**; **Supplementary Figure S1**) while maintaining an approximate 4-min WIT reduction. While these analyses confirm that the association was not driven solely by temporal progression, unmeasured residual confounding cannot be completely excluded.

The operative rationale for this association is consistent with the proposed membrane anatomy framework. By deconstructing the operative workflow into four reproducible technical nodes (fascial-plane entry, **Figure 1B**; pre-clamping hilar navigation, **Figure 1C**; pseudocapsule-guided excision, **Figures 1D, F**; and low-pressure check), the pathway enables surgeons to complete essential anatomical preparation before vascular occlusion (**Figure 1A**). This streamlined pre-clamping workflow provides a plausible anatomical mechanism for shorter WIT and compressed ischemia distributions (**Figures 2**, **3**), while also offering a structured framework for surgical teaching (14). In concordance with the primary endpoint, membrane-guided procedures demonstrated higher adjusted odds of achieving WIT ≤ 25 min and modified Trifecta (**Tables 2**; **Supplementary Table S2**). Positive surgical margins and severe complications remained rare in both groups (**Supplementary Table S2**), indicating that clamping-phase efficiency gains were achieved without compromising oncological or perioperative safety.

The clinical meaning of shorter WIT also warrants caution. While WIT reflects clamping-phase efficiency, shorter ischemia should not be directly equated with long-term renal functional preservation (2). Renal functional decline after partial nephrectomy is multifactorial and better predicted via dedicated risk models, such as the Martini nomogram (15). In our cohort, early postoperative renal functional changes did not differ materially between groups (**Table 2**). Thus, our results support WIT primarily as a process-efficiency metric under controlled hilar clamping rather than definitive evidence of long-term nephron preservation, nor do they support the indiscriminate pursuit of zero-ischemia surgery.

Several limitations should be noted. First, the retrospective, single-surgeon, single-platform design limits generalizability across institutions and experience levels. Second, while gradual pathway adoption was addressed via spline adjustments, overlap weighting, and temporal matching (**Supplementary Table S3**), residual unmeasured temporal confounding cannot be fully excluded. Third, pathway assignment relied on contemporaneous operative records without blinded video adjudication across the entire cohort. Fourth, individual RENAL score subcomponents were not captured as discrete structured variables. Fifth, step-specific bleeding during low-pressure inspection was not prospectively tracked. Sixth, 12-month eGFR data had substantial missingness (29.8% available), precluding robust long-term functional comparisons. Finally, the broader 342-case cohort was exploratory, and the lack of significant pathway-by-platform interaction (**Supplementary Table S4**) does not prove cross-platform equivalence.

## Conclusion

In summary, in this single-surgeon RAPN cohort with gradual and chronologically overlapping adoption, the membrane anatomy-guided pathway was associated with shorter WIT and improved clamping-phase efficiency. This association remained stable after rigorous adjustment for cumulative experience, calendar time, and measured clinical confounders. These findings support the procedural feasibility and efficiency of the membrane anatomy framework; however, prospective multicenter studies with standardized operative definitions and longitudinal functional follow-up are warranted to confirm causality.

## Data Availability

The data that support the findings of this study are available from the corresponding author upon reasonable request.
