# Supplementary material for: Membrane anatomy-guided robot-assisted partial nephrectomy: operational definition and association with warm ischemia time in a single-surgeon cohort
Source: Front Oncol. 2026 Sep 24;16:1953453. doi: 10.3389/fonc.2026.1953453 (PMC13656035; doi:10.3389/fonc.2026.1953453)
Supplement: Supplementary file 1 [file SupplementaryFile1.docx]

**Table S1. Covariate balance and weight diagnostics for propensity-score overlap weighting in the primary robot-assisted partial nephrectomy (RAPN) cohort.**

**(A) Covariate balance before and after overlap weighting**

| **Variable** | **Unweighted SMD** | **Overlap-weighted SMD** |
| --- | --- | --- |
| Age, years | 0.065 | 0.000 |
| Male sex | 0.277 | 0.000 |
| BMI, kg/m² | -0.085 | 0.000 |
| Tumor maximum diameter, cm | -0.010 | 0.000 |
| Preoperative eGFR, mL/min/1.73 m² | -0.327 | 0.000 |
| RENAL score | -0.248 | 0.000 |
| MAP score | -0.550 | 0.000 |
| Transperitoneal approach | 0.101 | 0.000 |
| ICG use | 0.227 | 0.000 |
| Cumulative RAPN case number | 0.472 | 0.000 |
| Case-number spline basis 1 | 0.344 | 0.000 |
| Case-number spline basis 2 | 0.416 | 0.000 |
| Case-number spline basis 3 | 0.222 | 0.000 |

**(B) Overlap-weight diagnostics and effective sample size**

| **Pathway** | **n** | **Minimum** | **Median** | **95th percentile** | **Maximum** | **Effective sample size** |
| --- | --- | --- | --- | --- | --- | --- |
| Overall | 151 | 0.009 | 0.137 | 0.842 | 0.969 | 74.9 |
| Conventional | 30 | 0.166 | 0.632 | 0.924 | 0.969 | 26.3 |
| Membrane anatomy-guided | 121 | 0.009 | 0.111 | 0.491 | 0.678 | 65.0 |

BMI, body mass index; eGFR, estimated glomerular filtration rate; ICG, indocyanine green; MAP, Mayo Adhesive Probability; RAPN, robot-assisted partial nephrectomy; RENAL, RENAL nephrometry score; SMD, standardized mean difference. Covariate balance was assessed using standardized mean differences, with |SMD| ≤ 0.10 denoting acceptable balance. Overlap weighting achieved exact numerical mean balance across all included clinical and chronological terms (weighted SMDs round to 0.000). Effective sample size represents the equivalent number of unweighted observations after weighting.

**Table S2. Chronology-adjusted multivariable logistic regression analyses of efficiency endpoints and descriptive incidence of rare safety events in the primary robot-assisted partial nephrectomy (RAPN) cohort.**

**(A) Pathway effect summary on binary efficiency endpoints**

| **Outcome** | **n** | **Events** | **Adjusted OR** | **95% CI** | ***P*** |
| --- | --- | --- | --- | --- | --- |
| WIT ≤ 25 min | 151 | 107 | 5.25 | 1.74 to 15.85 | 0.003 |
| Modified Trifecta | 151 | 105 | 4.97 | 1.63 to 15.13 | 0.005 |

**(B) Multivariable model for WIT ≤ 25 min**

| **Variable** | **Coefficient** | **Robust SE** | **OR** | **95% CI** | ***P*** |
| --- | --- | --- | --- | --- | --- |
| Intercept | 2.966 | 2.030 | 19.42 | 0.36 to 1038.86 | 0.144 |
| Membrane anatomy-guided pathway | 1.659 | 0.563 | 5.25 | 1.74 to 15.85 | 0.003 |
| Case-number spline basis 1 | -0.400 | 0.673 | 0.67 | 0.18 to 2.51 | 0.553 |
| Case-number spline basis 2 | -0.627 | 0.647 | 0.53 | 0.15 to 1.90 | 0.332 |
| Case-number spline basis 3 | -0.057 | 0.807 | 0.94 | 0.19 to 4.59 | 0.943 |
| Age, per year | -0.009 | 0.015 | 0.99 | 0.96 to 1.02 | 0.572 |
| Male sex | -0.589 | 0.503 | 0.55 | 0.21 to 1.49 | 0.242 |
| BMI, kg/m² | 0.003 | 0.042 | 1.00 | 0.92 to 1.09 | 0.944 |
| Tumor maximum diameter, cm | -0.277 | 0.099 | 0.76 | 0.62 to 0.92 | 0.005 |
| Preoperative eGFR, mL/min/1.73 m² | 0.010 | 0.011 | 1.01 | 0.99 to 1.03 | 0.342 |
| RENAL score | -0.263 | 0.121 | 0.77 | 0.61 to 0.97 | 0.030 |
| MAP score | -0.223 | 0.277 | 0.80 | 0.46 to 1.38 | 0.422 |
| Transperitoneal approach | -0.887 | 0.442 | 0.41 | 0.17 to 0.98 | 0.045 |
| ICG use | 0.473 | 0.585 | 1.60 | 0.51 to 5.05 | 0.419 |

**(C) Multivariable model for modified Trifecta**

| **Variable** | **Coefficient** | **Robust SE** | **OR** | **95% CI** | ***P*** |
| --- | --- | --- | --- | --- | --- |
| Intercept | 3.674 | 2.029 | 39.41 | 0.74 to 2101.42 | 0.070 |
| Membrane anatomy-guided pathway | 1.604 | 0.568 | 4.97 | 1.63 to 15.13 | 0.005 |
| Case-number spline basis 1 | -0.647 | 0.677 | 0.52 | 0.14 to 1.98 | 0.340 |
| Case-number spline basis 2 | -0.553 | 0.642 | 0.58 | 0.16 to 2.03 | 0.389 |
| Case-number spline basis 3 | -0.015 | 0.826 | 0.98 | 0.20 to 4.97 | 0.985 |
| Age, per year | -0.016 | 0.016 | 0.98 | 0.95 to 1.01 | 0.298 |
| Male sex | -0.636 | 0.504 | 0.53 | 0.20 to 1.42 | 0.207 |
| BMI, kg/m² | 0.005 | 0.041 | 1.01 | 0.93 to 1.09 | 0.903 |
| Tumor maximum diameter, cm | -0.272 | 0.097 | 0.76 | 0.63 to 0.92 | 0.005 |
| Preoperative eGFR, mL/min/1.73 m² | 0.009 | 0.011 | 1.01 | 0.99 to 1.03 | 0.377 |
| RENAL score | -0.294 | 0.123 | 0.75 | 0.59 to 0.95 | 0.016 |
| MAP score | -0.242 | 0.272 | 0.78 | 0.46 to 1.34 | 0.372 |
| Transperitoneal approach | -1.027 | 0.451 | 0.36 | 0.15 to 0.87 | 0.023 |
| ICG use | 0.483 | 0.589 | 1.62 | 0.51 to 5.14 | 0.412 |

**(D) Rare safety endpoints**

| **Outcome** | **n** | **Events** | **Conventional (n = 30)** | **Membrane anatomy-guided (n = 121)** | ***P*** |
| --- | --- | --- | --- | --- | --- |
| Positive surgical margin | 151 | 2 | 0 (0.0%) | 2 (1.7%) | 1.000 |
| Clavien-Dindo grade ≥ III | 151 | 3 | 0 (0.0%) | 3 (2.5%) | 1.000 |

BMI, body mass index; CI, confidence interval; df, degrees of freedom; eGFR, estimated glomerular filtration rate; HC3, heteroskedasticity-consistent covariance estimator 3; ICG, indocyanine green; MAP, Mayo Adhesive Probability; OR, odds ratio; RAPN, robot-assisted partial nephrectomy; RENAL, RENAL nephrometry score; SE, standard error; WIT, warm ischemia time. Multivariable logistic regression models utilized HC3 robust standard errors, adjusting for baseline clinical covariates and cumulative RAPN case sequence modeled via a centered natural cubic spline (df = 3). OR > 1.00 indicates higher odds in the membrane anatomy-guided pathway. Binary covariates are coded as male sex, transperitoneal approach, and ICG use. Rare safety endpoints were evaluated using Fisher's exact test without multivariable modeling due to sparse event counts. *P* < 0.05 are considered statistically significant.

**Table S3. Temporally adjacent matched analysis and post-matching covariate balance in the calendar-overlap cohort.**

**(A) Matching summary and paired outcome analysis**

| **Parameter** | **Result** |
| --- | --- |
| Calendar-overlap cohort cases | 22 conventional; 119 membrane anatomy-guided |
| Matched pairs | 22 pairs (1:1 matching without replacement) |
| Unmatched conventional cases | 0 |
| Date matching constraint | ≤ 30 absolute calendar days (median difference: 1.0 day [IQR 0.0–5.5]; maximum: 18 days) |
| Paired mean WIT difference | -4.05 min (95% CI -7.41 to -0.68) |
| Paired t-test *P* | 0.021 |
| Wilcoxon signed-rank test *P* | 0.020 |
| Maximum absolute matched-sample SMD | 0.676 |

**(B) Matched-sample baseline balance**

| **Variable** | **Conventional (n = 22)** | **Membrane anatomy-guided (n = 22)** | **SMD** |
| --- | --- | --- | --- |
| Age, years | 51.23 ± 14.85 | 48.68 ± 14.11 | -0.180 |
| Male sex | 10 (45.5%) | 13 (59.1%) | 0.276 |
| BMI, kg/m² | 25.37 ± 5.90 | 26.06 ± 4.82 | 0.131 |
| Tumor maximum diameter, cm | 3.77 ± 2.15 | 3.83 ± 2.70 | 0.025 |
| Preoperative eGFR, mL/min/1.73 m² | 106.15 ± 23.46 | 99.00 ± 15.43 | -0.369 |
| RENAL score | 7.41 ± 1.99 | 6.91 ± 2.00 | -0.257 |
| MAP score | 1.32 ± 0.78 | 0.77 ± 0.87 | -0.676 |
| Transperitoneal approach | 6 (27.3%) | 11 (50.0%) | 0.480 |
| ICG use | 7 (31.8%) | 8 (36.4%) | 0.096 |
| Calendar days from first RAPN | 695.36 ± 254.96 | 695.18 ± 255.73 | -0.001 |
| Cumulative RAPN case number | 78.77 ± 42.67 | 79.00 ± 42.63 | 0.005 |

**(C) Prespecified matching algorithm**

| **Parameter** | **Protocol design** |
| --- | --- |
| Target cohort | Calendar-overlap period only (cases 9–149) |
| Caliper constraint | ≤ 30 absolute calendar days (without replacement) |
| Optimization objective | Maximum cardinality, minimum total lexicographic cost assignment |
| Tie-breaking priority order | Absolute date difference, RENAL score, tumor diameter, MAP score, case number |
| Excluded variables | WIT and all postoperative outcomes strictly prohibited from matching |

BMI, body mass index; CI, confidence interval; eGFR, estimated glomerular filtration rate; ICG, indocyanine green; IQR, interquartile range; MAP, Mayo Adhesive Probability; RAPN, robot-assisted partial nephrectomy; RENAL, RENAL nephrometry score; SD, standard deviation; SMD, standardized mean difference; WIT, warm ischemia time. Continuous variables are presented as mean ± SD or median [IQR]; binary variables are presented as n (%). Baseline balance was assessed using standardized mean differences (|SMD| > 0.10 denotes meaningful imbalance). Matching was performed as a sensitivity analysis for temporal proximity and does not establish complete confounding control. *P* < 0.05 are considered statistically significant.

**Table S4. Primary and exploratory analyses in the broader minimally invasive partial nephrectomy cohort (n = 342).**

**(A) Sensitivity models across the full minimally invasive cohort**

| **Model specification** | **n** | **Difference in WIT (min)** | **95% CI** | ***P*** | **Chronology and platform adjustment** |
| --- | --- | --- | --- | --- | --- |
| Case-number spline (HC3) | 342 | -4.86 | -6.98 to -2.74 | < 0.001 | Centered natural cubic spline of total case sequence (df = 3) |
| Calendar-time spline (HC3) | 342 | -4.37 | -6.47 to -2.26 | < 0.001 | Centered natural cubic spline of calendar days (df = 3) |
| Median regression | 342 | -3.81 | -6.16 to -1.46 | 0.002 | Centered natural cubic spline of total case sequence (df = 3) |
| Chronology- and platform-informed overlap weighting | 342 | -4.87 | -7.15 to -2.59 | < 0.001 | PS model with case-number spline; effective sample size = 114.6 |

**(B) Exploratory surgical pathway-by-platform interaction model**

| **Model term** | **Difference in WIT (min)** | **95% CI** | ***P*** | **Clinical interpretation** |
| --- | --- | --- | --- | --- |
| Pathway × platform interaction | 0.34 | -3.83 to 4.50 | 0.874 | Heterogeneity of pathway effect between RAPN and LPN |
| Pathway contrast within LPN stratum | -5.07 | -8.44 to -1.71 | 0.003 | Adjusted membrane anatomy-guided vs. conventional difference in LPN |
| Pathway contrast within RAPN stratum | -4.74 | -7.38 to -2.09 | < 0.001 | Adjusted membrane anatomy-guided vs. conventional difference in RAPN |

CI, confidence interval; df, degrees of freedom; HC3, heteroskedasticity-consistent covariance estimator 3; LPN, laparoscopic partial nephrectomy; PS, propensity score; RAPN, robot-assisted partial nephrectomy; WIT, warm ischemia time. Difference in WIT is calculated as membrane-guided minus conventional surgery (negative values indicate shorter WIT in the membrane-guided pathway). All broader-cohort models adjusted for surgical platform, chronological progression, and baseline clinical covariates. The interaction model additionally included a pathway-by-platform product term to evaluate statistical heterogeneity across surgical platforms. Results are exploratory and associational, without overriding the primary RAPN analysis. *P* < 0.05 are considered statistically significant.

**Figure S1. Standardized mean differences (SMDs) of covariates before and after propensity-score overlap weighting in the primary robot-assisted partial nephrectomy (RAPN) cohort.** Absolute SMDs are plotted for baseline demographic, clinical, tumor, and chronological covariates before weighting (unweighted, red circles) and after overlap weighting (overlap-weighted, green squares). The vertical dashed line indicates the prespecified balance threshold of |SMD| = 0.10. Corresponding balance statistics are detailed in Table S1. BMI, body mass index; eGFR, estimated glomerular filtration rate; ICG, indocyanine green; MAP, Mayo Adhesive Probability; RAPN, robot-assisted partial nephrectomy; RENAL, RENAL nephrometry score; SMD, standardized mean difference.

**Figure S2. Propensity-score distributions according to operative pathway before and after overlap weighting.** Kernel density plots illustrate the distribution of propensity scores for the conventional pathway (red curves, n = 30) and the membrane anatomy-guided pathway (green curves, n = 121) before weighting (unweighted, *left*) and after propensity-score overlap weighting (overlap-weighted, *right*).

**Figure S3. Cumulative sum (CUSUM) analysis of warm ischemia time (WIT) across the chronological robot-assisted partial nephrectomy (RAPN) series.** The dark green solid line displays the unadjusted cumulative sum of deviations from the cohort mean WIT plotted against consecutive RAPN case numbers (cases 1 to 151). The vertical dashed grey line marks the peak deviation at case 21, indicating the descriptive transition of procedural efficiency. Bottom tick marks (rug plot) denote individual surgical pathways: conventional pathway (pink vertical ticks, n = 30) and membrane anatomy-guided pathway (green vertical ticks, n = 121). CUSUM, cumulative sum; RAPN, robot-assisted partial nephrectomy; WIT, warm ischemia time.
